# Three-dimensional cultured mesenchymal stem cells enhance repair of ischemic stroke through inhibition of microglia

**DOI:** 10.1186/s13287-021-02416-4

**Published:** 2021-06-21

**Authors:** Yuejiao Li, Yankai Dong, Ye Ran, Yanan Zhang, Boyao Wu, Jundong Xie, Yanpei Cao, Miaohua Mo, Sen Li, Hao Deng, Wenbo Hao, Shengyuan Yu, Yaojiong Wu

**Affiliations:** 1 State Key Laboratory of Chemical Oncogenomics, and Shenzhen Key Laboratory of Health Sciences and Technology, Tsinghua Shenzhen International Graduate School, Tsinghua University, Shenzhen, 518055 China; 2 School of Life Sciences, Tsinghua University, Beijing, 100084 China; 3 Department of Neurology, The Chinese PLA General Hospital, Medical School of Chinese PLA, Beijing, 100853 China; 4 Tsinghua-Berkeley Shenzhen Institute (TBSI), Tsinghua University, Shenzhen, 518055 China; 5 Peking University Shenzhen Hospital, Shenzhen, 518036 China; 6 Institute of Antibody Engineering, School of Laboratory Medicine and Biotechnology, Southern Medical University, Guangzhou, 510515 China; 7 Department of Neurology, The Chinese PLA General Hospital, Beijing, 100853 China

**Keywords:** Mesenchymal stem cells, Ischemic stroke, Microglia, Mincle, Neuroinflammation

## Abstract

**Background:**

We show previously that three-dimensional (3D) spheroid cultured mesenchymal stem cells (MSCs) exhibit reduced cell size thus devoid of lung entrapment following intravenous (IV) infusion. In this study, we determined the therapeutic effect of 3D-cultured MSCs on ischemic stroke and investigated the mechanisms involved.

**Methods:**

Rats underwent middle cerebral artery occlusion (MCAO) and reperfusion. 1 × 10^6^ of 3D- or 2D-cultured MSCs, which were pre-labeled with GFP, were injected through the tail vain three and seven days after MCAO. Two days after infusion, MSC engraftment into the ischemic brain tissues was assessed by histological analysis for GFP-expressing cells, and infarct volume was determined by MRI. Microglia in the lesion were sorted and subjected to gene expressional analysis by RNA-seq.

**Results:**

We found that infusion of 3D-cultured MSCs significantly reduced the infarct volume of the brain with increased engraftment of the cells into the ischemic tissue, compared to 2D-cultured MSCs. Accordingly, in the brain lesion of 3D MSC-treated animals, there were significantly reduced numbers of amoeboid microglia and decreased levels of proinflammatory cytokines, indicating attenuated activation of the microglia. RNA-seq of microglia derived from the lesions suggested that 3D-cultured MSCs decreased the response of microglia to the ischemic insult. Interestingly, we observed a decreased expression of *mincle*, a damage-associated molecular patterns (DAMPs) receptor, which induces the production of proinflammatory cytokines, suggestive of a potential mechanism in 3D MSC-mediated enhanced repair to ischemic stroke.

**Conclusions:**

Our data indicate that 3D-cultured MSCs exhibit enhanced repair to ischemic stroke, probably through a suppression to ischemia-induced microglial activation.

**Supplementary Information:**

The online version contains supplementary material available at 10.1186/s13287-021-02416-4.

## Background

Ischemic stroke is the first leading cause of death and neurological dysfunction in China [1]. Currently, only intravenous thrombolysis with tissue plasminogen activator and mechanical thrombectomy have shown to be effective in the acute phase of ischemic stroke. However, these approaches are not promptly accessible to most patients due to a narrow therapeutic window [2]. There has been no effective drug to ameliorate sensorimotor functions except for rehabilitation, leaving most of the patients suffering from life-long disability. In this sense, cell therapy has emerged as a promising therapy for stroke in the subacute phase. Mesenchymal stem cells (MSCs) have attracted much attention due to advantages over other types of cells, including absence of ethical and legal concerns, low immunogenicity, and sufficient availability [3–5]. In preclinical and clinical studies, the non-invasive intravenous (IV) route is the most commonly used approach for MSCs delivery [6]. Many literatures have shown that IV administration of MSCs promoted tissue repair and had a therapeutic effect on ischemic stroke because of its multiple differentiation potential, anti-inflammatory and immunomodulatory properties, and expression of trophic factors and cytokines [7–9]. However, the loss of homing and engraftment potential in cultured MSCs after expanding under conventional two-dimensional (2D) culture conditions has warranted the development of novel clinical strategies [10]. Therefore, efforts need to be made to improve the homing ability of culture expanded MSCs after cell transplantation.

Our and others’ previous studies have shown that three-dimensional (3D) culture significantly reduced the size of MSCs compared with 2D monolayer culture, thus reducing the vascular obstructions in the lungs after IV injection [11–15]. Besides, compared with 2D MSCs, 3D MSCs exhibited decreased expression of integrins, as excess expression of integrins after 2D culture is a cause for MSC entrapment in the lung [15]. The escape of MSCs from the lung allows the cells to circulate in the blood and therefore distribute more efficiently into other tissues [13, 15]. Furthermore, studies have shown that the expression of homing receptors, such as CXCR4, is usually decreased on the surface of culture-expanded MSCs [16]. 3D culture restores the expression levels of CXCR4 in MSCs and improves their homing capacity to the injury sites after systematic administration [13, 16–18].

It has been suggested that inflammation plays an important role in the pathogenesis of ischemic stroke [19]. As resident macrophage cells, microglia are the principal immune cells of the brain, and the first to respond to the pathophysiological changes induced by ischemic stroke [19]. In the early stages of stroke, classically activated microglia contribute to neuronal damage and exacerbate tissue injury by producing inflammatory cytokines and cytotoxic substances [20]. In vivo studies have shown that MSC administration significantly reduced microglia activation, as indicated by decreased immunoreactivity of ED1 and ionized calcium-binding adapter molecule 1 (Iba1) [21]. Pre-conditioning of MSCs under 3D environments prior to transplantation increased their anti-inflammatory properties by secreting more immunoregulatory factors such as prostaglandin E2, transforming growth factor beta 1, interleukin-6 (IL-6), tumor necrosis factor-stimulated gene-6 (TSG-6), hepatocyte growth factor (HGF), and stanniocalcin 1 (STC1) [13, 22–25]. In vitro study has demonstrated that 3D MSCs reduced the macrophages activation and promoted the transition of stimulated macrophages from a proinflammatory M1 phenotype to a more anti-inflammatory phenotype [13, 23]. Another study showed a decreased infiltration of macrophages with 3D-exosomes treatment in the cisplatin-induced murine acute kidney injury model [26]. However, the immunoregulatory effects of 3D MSCs in the ischemic stroke animal model are not elucidated, especially on the modulation of microglia.

In this study, we found that after IV infusion, 3D MSCs exhibited enhanced homing ability to the ischemic lesion, reduced the infarct volume, and restored the neurological dysfunctions in rats with ischemic stroke, compared to 2D MSCs. In addition, 3D MSC treatment reduced the activation of microglia with decreased immunoreactivity of Iba1.

## Methods

### Animals

Male Sprague-Dawley rats, average weighing 280–320 g were obtained from the Laboratory Animal Center, Guangdong Province, China. All of the animals were maintained in cages at a temperature-controlled environment, with a 12-h light-dark cycle and free access to food and fresh water. The procedures involving animal and their care were conducted under the approval of the Ethics Committee of Tsinghua University.

### Middle cerebral artery occlusion model

Middle cerebral artery occlusion (MCAO) was induced in male Sprague-Dawley rats as described in our previous studies [27]. Briefly, animals were anesthetized with a mixture of 1 to 2% isoflurane in nitric oxide/oxygen via a face mask. The body temperature was maintained at 37 °C during the surgical procedures. A midline cervical incision was made with subsequent exposure of the left common carotid artery, internal carotid artery, and external carotid artery. The common carotid artery and external carotid artery were ligated with a 4–0 silk. Thereafter, a silicon-rubber-coated round-tip nylon suture was advanced from the common carotid artery until it blocked the origin of the middle cerebral artery. After 120 min of MCAO, animals were reanesthetized and reperfused by withdrawing the nylon suture.

### Cell culture

MSCs were isolated from human placenta as previously described [28]. Cells were culture expanded in Dulbecco’s modified Eagle’s medium (DMEM; Corning) supplemented with 10% fetal bovine serum (FBS; Biological Industries) at 37 °C with 5% CO_2_. Spheroid culture of MSCs were performed by culturing MSCs in cell culture spinner flasks in Minimum Essential Medium Eagle (MEM, Sigma) supplemented with 5% EliteGro™-Adv (Biomedical EliteCell Corp.) for 60 h at 37 °C with 5% CO_2_. To obtain single cells from spheroids, spheroids were incubated with 0.25% trypsin/EDTA at 37 °C for 4–6 min with gentle pipetting every 2–3 min.

### Intravenous administration of MSCs

The animals were randomly divided into the following three experimental groups after MCAO: PBS, 2D MSCs, and 3D MSCs. In one set of experiments, the rats were slowly infused with 1 × 10^6^ cells/200 μl PBS into the tail vein at 3 days and 7 days after MCAO. The same amount of PBS was injected as the control. The animals were sacrificed at 2 weeks after stroke. In another set of experiments, 3 × 10^6^ 2D or 3D MSCs in 200 μl PBS were intravenously injected 3 days after MCAO as mentioned above. The rats were sacrificed at 2 days after cell transplantation. To investigate the viability and engraftment of MSCs after infusion, cells were labeled using a green fluorescent protein (GFP) lentivirus (Cyagen) according to the manufacturer’s instructions. Cells were filtered through a 40-μm cell strainer to generate a single cell suspension before transplantation.

### Behavioral testing

The behavioral tests were performed with modified neurological severity score (mNSS) and adhesive-removal test at 1, 5, 9, and 13 days post-MCAO as previously described [29, 30]. mNSS test was used to evaluate the neuromotoric capacity of the animals. Motor, sensory, balance, and reflex tests are included and are graded on a scale of 0 to 18 (0 for normal score; 18 for maximal deficit score). For adhesive-removal tests, round dots of adhesive tape were used as bilateral tactile stimuli, occupying the distal-radial region of each forelimb wrist. The time, to a maximum of 120 s, for each rat to remove the right paper dots was recorded. Three trials were conducted daily, with an interval of at least five minutes. Data are presented as absolute values in seconds (adhesive-removal test) or score points (mNSS test).

### Immunofluorescence

Rats were anesthetized with sodium pentobarbital (30 mg/kg, i.p.) and perfused with 100 mL saline and 100 mL 4% PFA. Tissues were dissected and fixed in 4% PFA at 4 °C overnight. Fixed samples were dehydrated with 10%, 20%, and 30% sucrose successively and embedded in OCT. Frozen sections of 10-μm thickness were cut through the entire forebrain. Several consecutive sections of the entire brain were selected and processed for immunofluorescent staining with the appropriate primary antibody at 4 °C overnight against NeuN (a neuronal marker; Millipore, MAB377; 1:100), GFAP (a marker of astrocytes; Cell Signaling Technology, 12389; 1:200), and Iba1 (a microglia marker; Abcam, ab178846; 1:200). Matching fluorescence-conjugated secondary antibodies from Jackson ImmunoResearch were employed. Nuclei were counterstained with DAPI. All samples were analyzed with a confocal laser scanning microscope (FV1000, Olympus, Japan).

### Quantitative reverse transcription polymerase chain reaction (qRT-PCR) analysis

Total RNA was extracted from the fresh brain tissues and MSCs using the RNAiso Plus reagent (Takara, 9109), according to the manufacturer’s instructions. RNA was quantified using Nanodrop (Thermo Scientific). cDNA was synthesized from 2 μg of total RNA with Hifair II 1st Strand cDNA Synthesis SuperMix for qPCR (Yeasen, 11123ES60). Next, qRT-PCR was performed on 7300 Real Time PCR System (Applied Biosystems) using Hieff qPCR SYBR Green Master Mix (High Rox Plus, Yeasen, 11203ES08) in triplicate. The relative expression of target genes was analyzed according to the 2^−ΔΔCT^ method and normalized to the level of GAPDH. All primer sequences were shown in Supplementary Table S1.

### Fluorescence-activated cell sorting

At 2 days after cell infusion, rats were deeply anesthetized with sodium pentobarbital (30 mg/kg, i.p.) and subjected to transcardial perfusion with 200 mL sterile ice-cold saline. After decapitation, each rat brain was harvested and the diseased hemispheres were stored in HBSS (without calcium, magnesium) (Thermo Fisher Scientific, C14175500BT). Isolated brain tissues were sliced into small pieces using a sterile scalpel on ice and enzymatically homogenized at 30 °C for 30 min with a pre-heated enzyme mix containing papain (100 U, Worthington, LS003119), dispase II (6 U, Sigma-Aldrich D4693), and DNAse I (100 U, Sigma-Aldrich D5025). Prior to use, papain was activated for 30 min at 37 °C and 5% CO_2_. To stop all digestions, samples were diluted with cold HBSS and placed on ice. Solutions were then further homogenated using Pasteur pipette with the fire-polished tip for 10 times in approximately 45 s and filtered through a 70-μm cell strainer (BD, 352350) to remove large debris and cell clusters. The resulting single cell suspension was centrifuged at 300 g for 10 min at RT. Myelin and debris were removed using Percoll (Yeasen, 40501ES60) density gradient centrifugation. Briefly, a single suspension of cells was mixed with 9 mL of 30% isotonic Percoll solution and centrifugated at 300×*g* for 30 min, at 18 °C. After centrifugation, cells were collected from the bottom layer of the 30% Percoll gradient, washed by adding 1 × HBSS and centrifugated for 10 min at 300×*g*. The cells were resuspended with 1 mL DMEM. Following this, the resuspended cells were stained with trypan blue and cell counting was performed using a hemocytometer. Cells were pre-blocked with excess irrelevant purified Ig from same isotype as the antibodies used for immunofluorescent staining on ice for 10 min. Then, the cells were labeled for surface markers CD45 PE (202207, 1:100, BioLegend) and CD11b APC (201809, 1:100, BioLegend) in Cell Staining Buffer (420201, Biolegend) on ice for 30 min in the dark. Dead cells were excluded from analysis by labeling with 7-AAD Viability Staining Solution (40745ES64, Yeasen). CD45^low^CD11b^+^ microglia were sorted on SH800S Cell Sorter (Sony Biotechnology) and the data was analyzed with FlowJo software (Tree Star).

### RNA-sequencing

Total RNA was extracted from the isolated microglia from the brain using TRIzol reagent. Subsequently, the total RNA was identified and the full-length cDNA was amplified by PCR. The average molecule length was determined using the Agilent 2100 bioanalyzer instrument (Agilent High Sensitivity DNA Reagents). Library was constructed following Tagmentation-based library construction protocol. PCR products were purified and selected with the Agencourt AMPure XP-Medium kit. DNA was quantified by Agilent Technologies 2100 bioanalyzer. Library was qualified by the Agilent Technologies 2100 bioanalyzer. The library was amplified to make DNA nanoball (DNB) which had more than 300 copies of one molecular. The DNBs were loaded into the patterned nanoarray and single end 50 bases reads were generated in the way of sequenced by combinatorial Probe-Anchor Synthesis (cPAS). The raw sequencing data were aligned to the rat genome. Differentially expressed genes (DEGs) were identified by PossionDis analysis with FDR < 0.001.

### Microglial cell culture and activation

The BV2 mouse microglial cell line was bought from China Center for Type Culture Collection. Cells were culture expanded at 37 °C with 5% CO_2_ in DMEM (Corning, 10-017-CV) supplemented with 10% FBS (Biological Industries) and antibiotics. To assess the effects of 2D and 3D MSCs on BV2 cells, a total of 3 × 10^5^ BV2 cells were placed in a 6-well chamber and stimulated with 100 ng/ml LPS for 4 h. One hundred microliters of conditioned medium from 2D or 3D MSCs were added simultaneously. After 4 h of treatment, BV2 cells were harvested for RNA extraction.

### Statistical analysis

Statistical analysis of all experiments was carried out using GraphPad Prism 8.0 software. All data were presented as means ± SEM. Unpaired Student’s *t* tests were performed to compare differences between two groups. One-way analysis of variance was used to compare differences involving three or more groups. P value less than 0.05 was considered statistically significant.

## Results

### 3D spheroid culture reduces the size of MSCs

**Fig. 1:**
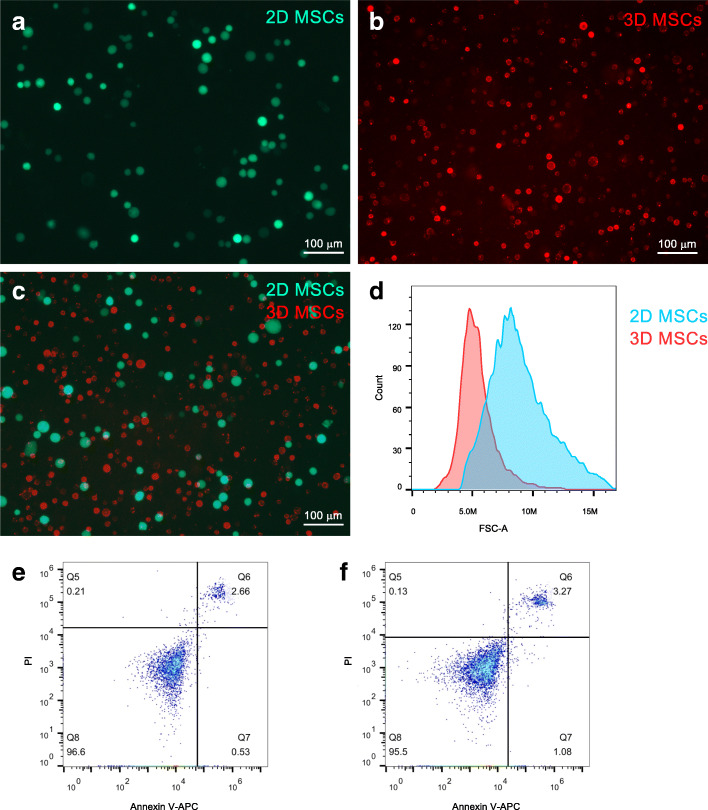
Three-dimensional (3D) spheroid culture reduced the size of mesenchymal stem cells (MSCs). Two-dimensional (2D) MSCs **a** and 3D MSCs **b** were labeled with GFP and DiI, respectively. Photos showed the size of MSCs. **c** Comparison of the size of 2D and 3D MSCs. **d** Histogram showed the size of 2D and 3D MSCs with flow cytometry. The apoptosis of 2D MSCs (**e**) and 3D MSCs (**f**) were analyzed with annexin V/PI staining

After 3D spheroids culture, the diameter of MSCs significantly decreased (Fig. 1A–D), which is consistent with our previous studies [11, 14, 27]. Importantly, annexin V/PI staining of the cells by flow cytometry analysis showed that the 3D spheroid culture did not increase cell apoptosis (Fig. 1E, F). This indicates that the 3D spheroid culture can reduce the size of MSCs without affecting cell viability.

### 3D MSC infusion decreases infarct volume and improves neurological function

**Fig. 2:**
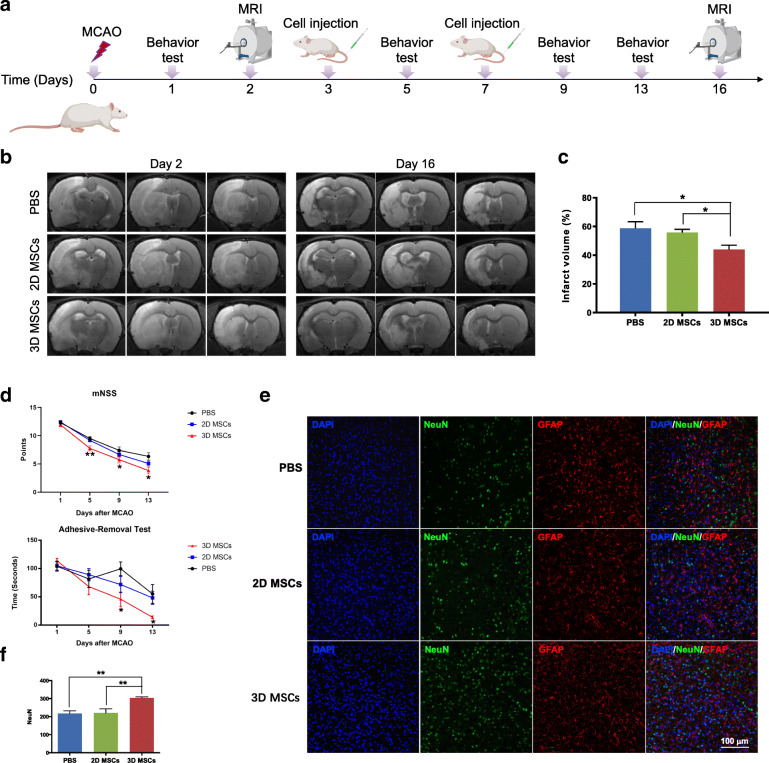
3D MSCs alleviated ischemia-induced injury. **a** The experimental timeline was illustrated. Rats were intravenously injected 3 and 7 days after middle cerebral artery occlusion (MCAO) with PBS, 2D and 3D MSCs, respectively. The infarct volume was measured by MRI at 2 and 16 days after stroke. Rats were subjected to modified neurological severity score (mNSS) and adhesive-removal test at 1, 5, 9, and 13 days after stroke. **b** Representative T2-weighted MRI in rats treated as above. **c** 16 days post-stroke, quantitative graphs of MRI showed a significant lower infarct volume in the 3D MSCs transplanted group compared with 2D MSCs and PBS group. Data are shown as mean ± SEM; n = 6 for PBS group; n = 9 for 2D MSCs group and n = 8 for 3D MSCs group. *p < 0.05. **d** Neurological function of rats was determined by mNSS test and adhesive-removal test. 3D MSC treatment significantly enhanced neurological functional recovery at 5, 9, and 13 days after stroke compared with PBS treatment. Data are shown as mean ± SEM; n = 11 for 2D MSCs group; n = 12 for PBS and 3D MSCs group. *p < 0.05 compared with PBS group. **e**, **f** Immunofluorescence imaging of neuronal nuclei (NeuN)-positive cells in the ischemic penumbra at 5 days after MCAO. The quantitative analysis indicated that the total number of NeuN-positive cells in the ipsilateral penumbra was greater in the 3D MSCs administered group than that in the PBS and 2D MSCs group. Data are shown as mean ± SEM; n = 3 for each group. *p < 0.05

In order to investigate the effects of 2D and 3D MSCs on the recovery of ischemic injury, MCAO rats were intravenously injected with PBS, 1 × 10^6^ of 2D or 3D MSCs at 3 and 7 days after MCAO, respectively (Fig. 2A). The lesion volume measured by MRI at 16 days post-stroke in 3D MSC-treated rats was significantly lower than that in the PBS group and the 2D MSCs group (Fig. 2B, C). Behavioral improvements were monitored on 1, 5, 9, and 13 days following MCAO. Neurological function was tested by mNSS before and after MSC treatment, demonstrating that injection of 3D MSCs significantly improved functional recovery compared to that of the PBS group at 5, 9, and 13 days after stroke. Whereas the neurological function in 2D MSC-treated rats was indistinguishable from the PBS group. To evaluate forelimb deficits, the impaired forelimb usage was further analyzed via the adhesive-removal test. Results showed that rats treated with 3D MSCs removed the tape significantly faster than rats treated with PBS (Fig. 2D). Together, the mNSS and the adhesive-removal test suggest that IV administration of 3D MSCs enhances sensorimotor outcomes after ischemic injury. To explain whether these functional improvements are due to neuronal survival, the expression of neuronal marker NeuN was additionally determined using immunofluorescence imaging at 5 days after MCAO. Results showed that the number of NeuN-positive neurons was significantly increased in 3D MSC-treated animals compared to the PBS- and 2D MSC-treated rats (Fig. 2E, F). These data demonstrated that systematic administration of 3D-cultured MSCs significantly improved the ischemic brain repair.

### 3D MSCs exhibits enhanced homing ability to the brain

**Fig. 3:**
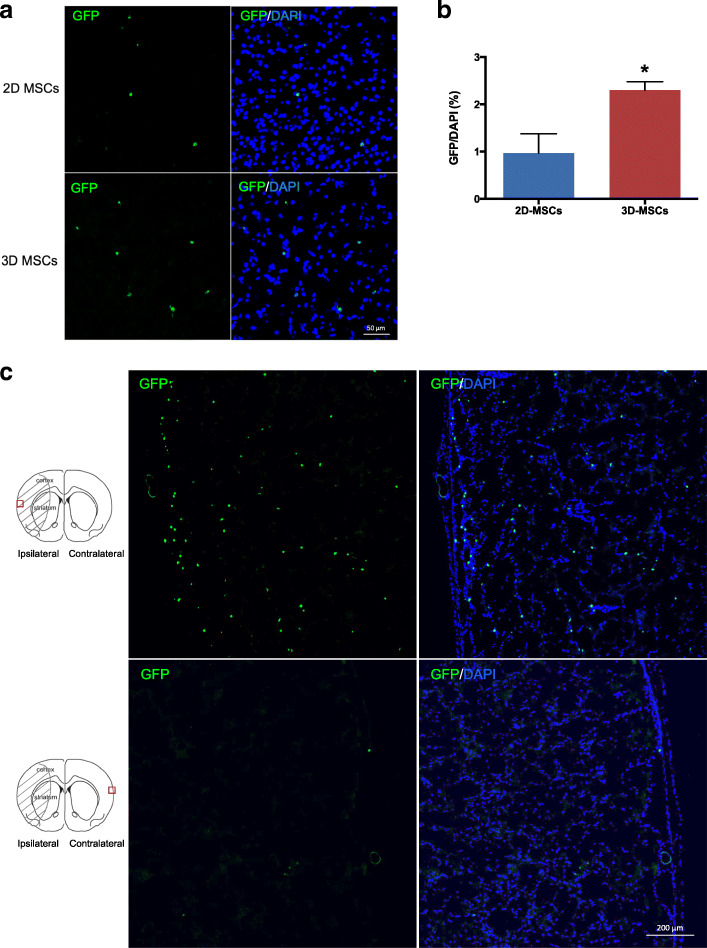
3D MSCs exhibited enhanced homing ability to ischemic brain tissues. **a**, **b** Immunofluorescence imaging of GFP-labeled MSCs in the ischemic brain at 2 days after IV injection. The quantitative graph showed that significantly more 3D MSCs were found in the ischemic core than 2D MSCs. Data are shown as mean ± SEM; n = 3 for each group. *p < 0.05. **c** Immunofluorescence imaging of GFP-labeled 3D MSCs in the diseased and the contralateral hemisphere. MSCs preferentially migrated to the ischemic hemisphere

As 3D culture was reported to restore the expression of homing receptors of MSCs [13], we determined the homing of 3D MSCs to the ischemic lesion with immunofluorescence imaging. Both GFP-labeled 2D and 3D MSCs were observed in the ischemic hemisphere at 2 days after infusion, whereas significantly more 3D MSCs were detected than 2D MSCs (Fig. 3A, B). Besides, compared with the contralateral hemisphere, most MSCs were observed in the ischemic hemisphere (Fig. 3C). These results suggest that 3D-cultured MSCs improved the neurological repair partly through their enhanced homing ability to the ischemic lesion.

### 3D MSCs ameliorate ischemia-induced microglial activation in vivo

**Fig. 4:**
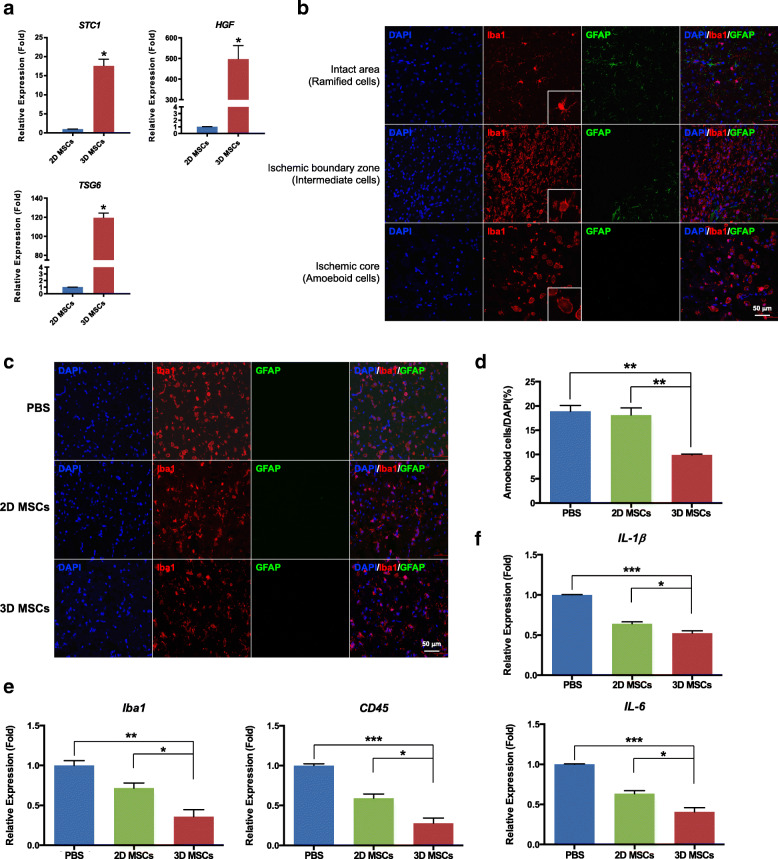
3D MSCs exhibited enhanced anti-inflammatory effects on microglia in vivo. **a** The expression of stanniocalcin 1 (*STC1*), hepatocyte growth factor (*HGF*), and tumor necrosis factor-stimulated gene-6 (*TSG-6*) between 2D and 3D MSCs was evaluated with qRT-PCR. **b** Different morphology of ionized calcium-binding adapter molecule 1 (Iba1)-positive microglia with magnified images at 5 days after ischemic stroke. **c** Immunofluorescence imaging of microglia and glial fibrillary acidic protein (GFAP)-positive astrocytes in the ischemic core at 5 days after MCAO. The number of amoeboid Iba1-positive cells was reduced in the 3D MSC-administered group. **d** The quantitative analysis of amoeboid Iba1-positive cells indicated that the number of amoeboid cells in the ischemic core was significantly decreased in the 3D MSC-administered group than that in the PBS and 2D MSCs group. Data are shown as mean ± SEM; n = 3 for each group. **p < 0.01. **e**, **f** qRT-PCR analysis showed that IV injection of 3D MSCs significantly decreased the expression of *Iba1*, *CD45*, interleukin-1β (IL-1β), and interleukin-6 (IL-6) compared with the PBS and 2D MSCs group. Data are shown as mean ± SEM; n = 3 for each group. *p < 0.05, **p < 0.01, ***p < 0.001

Spheroid culture of MSCs was reported to enhance the secretion of factors mediating inflammatory and immune responses [13, 31]. Here, we found the mRNA expression of *STC1*, *HGF*, and *TSG-6* was significantly increased in 3D-culured MSCs (Fig. 4A). The in vivo anti-inflammatory effects of 3D MSCs on microglia were further investigated with staining of microglia marker Iba1 in brain tissues at 2 days after cells infusion. Astrocyte marker glial fibrillary acidic protein (GFAP) was also stained to indicate the ischemic core, as astrocytes form glial scar in the perilesional area after ischemia [32]. Iba1-positive microglia were observed in both the intact and the ischemic hemisphere with different morphological appearances. Ramified cells with long thin processes were mainly localized in the intact area of the ischemic hemisphere and the contralateral hemisphere. Intermediate cells with swollen processes were mostly observed in the peri-infarct regions. Amoeboid cells with round shape and no processes were mainly detected in the ischemic core [33] (Fig. 4B). When stimulated, microglia gained morphological changes from a ramified shape to an amoeboid-shaped “activation” status [34]. As amoeboid microglia were shown to be abundant in the core area between 3 and 7 days after stroke, we compared the number of amoeboid microglia 5 days after MCAO. The number of amoeboid microglia in the ischemic core was significantly decreased in the 3D MSCs group compared to the PBS and 2D MSCs group as shown by the immunofluorescence staining (Fig. 4C, D). qRT-PCR results also demonstrated decreased expression of microglial markers *Iba1* and *CD45* in 3D MSCs group (Fig. 4E). The expression of proinflammatory cytokines interleukin-1β (IL-1β) and interleukin-6 (IL-6) was further determined and results showed a reduction of their expression in 3D MSCs group than PBS and 2D MSCs group (Fig. 4F). These data suggest enhanced anti-inflammatory effects of 3D MSCs on microglia after ischemic injury.

### Transcriptomic changes in microglia with MSC administration

**Fig. 5:**
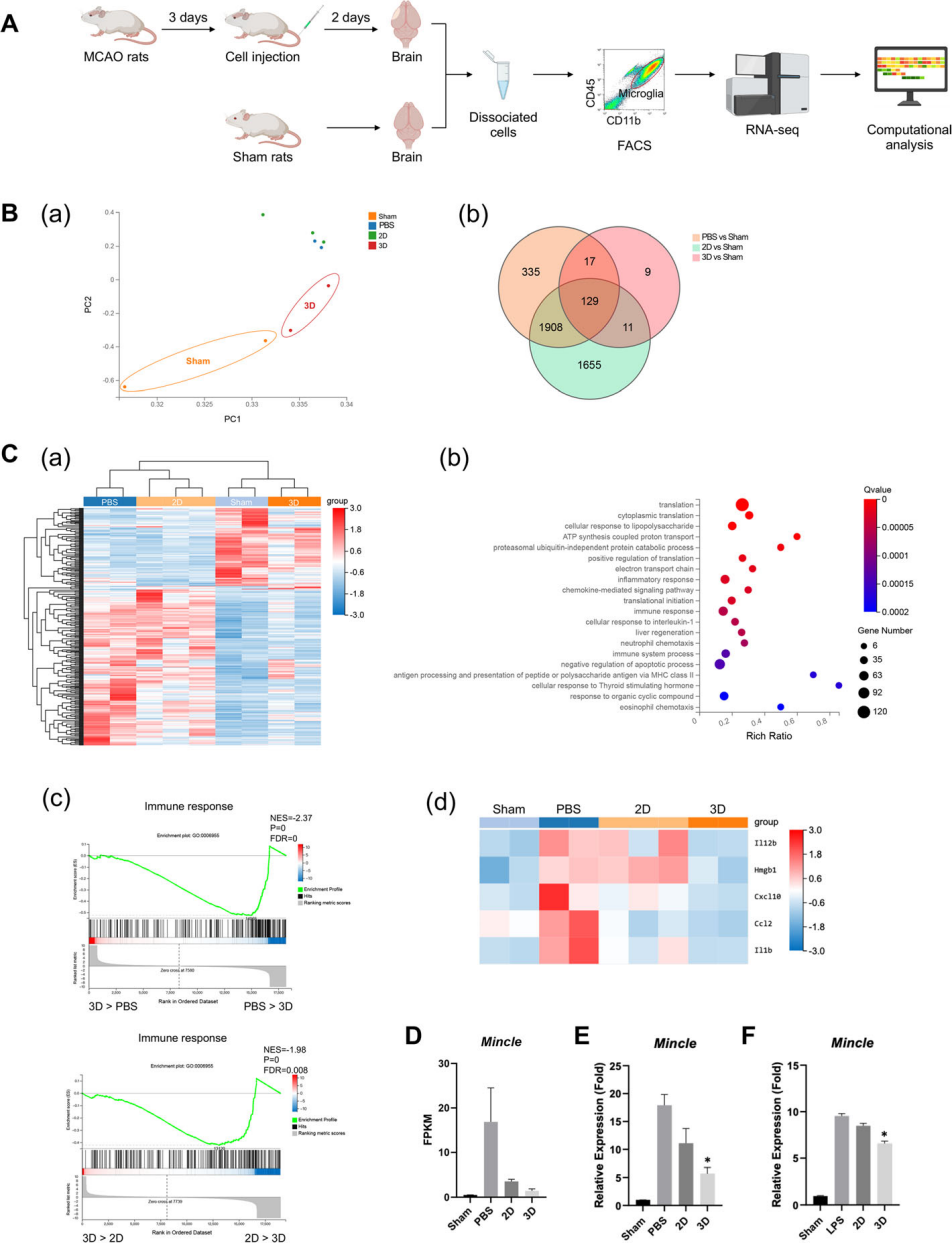
Transcriptomic changes in microglia with MSC administration. **A** A schematic diagram depicting sample preparation for RNA-seq. CD45^low^CD11b^+^ microglia were isolated from the brain of Sham rats or the ischemic hemisphere 2 days after cell infusion. Total RNA was extracted from isolated microglia for RNA-seq analysis. **B** PCA (a) of microglia population sampled from sham rats, PBS-, 2D MSC-, or 3D MSC-treated rats using PC1 and PC2 suggests clear separation of sham microglia and 3D MSC-treated microglia from PBS and 2D MSC-treated microglia. Venn diagram (b) showing the interactions among DEGs between Sham with PBS, 2D, or 3D MSC-treated samples. **C** Heatmap (a) showing the expression profiles of 341 genes which participate in the immune system. Gene Ontology (GO) biological process analysis (b) for the 530 downregulated genes by 3D MSC infusion compared with PBS and 2D MSC infusion showed genes involved in biological processes including cellular response to lipopolysaccharide, inflammatory responses, immune response, and immune system process. Gene set enrichment analysis (GSEA) (c) showing positive enrichment in transcriptome with treatment of PBS and 2D MSCs for immune response gene set. Heatmap (d) showing the expression profiles of genes belonging to proinflammatory mediators. **D** The expression of *Mincle* based on RNA-seq data. FPKM, fragments per kilobase of transcript per million mapped reads. **E** The expression of *Mincle* was validated with qRT-PCR. *p < 0.05 compared with PBS group. **F** The expression of *Mincle* was determined in LPS-activated microglia with treatment of 2D or 3D MSCs conditioned media. *p < 0.05 compared with LPS group

To elucidate the molecular changes in microglia with MSCs transplantation, CD45^low^CD11b^+^ microglia were isolated 2 days after cell administration for RNA-seq analysis (Fig. 5A, Supplemental Fig. 1). The principal component analysis (PCA) showed that the transcriptome of microglia from sham-operated rats (Sham-MG) and 3D MSC-infused rats (3D-MG) differ from the transcriptome of microglia from PBS (PBS-MG) or 2D MSC (2D-MG)-treated rats (Fig. 5Ba). The Venn diagram showed the DEGs between Sham-MG with PBS-MG, 2D-MG, or 3D-MG, which demonstrated the high similarity of Sham-MG and 3D-MG (Fig. 5Bb). DEGs were processed for KEGG pathway enrichment analysis and results showed enrichment of 341 genes for immune system. Heatmap of the expression of these genes showed the clustering of Sham-MG and 3D-MG (Fig. 5Ca). The 530 downregulated genes in 3D-MG compared with PBS-MG and 2D-MG were subjected to Gene Ontology (GO) biological process analysis. Results revealed the involvement of genes in biological processes including cellular response to LPS, inflammatory responses, immune response, and immune system process (Fig. 5Cb). Gene set enrichment analysis (GSEA) illustrated that immune response related genes were enriched among the DEGs in PBS-MG and 2D-MG (Fig. 5Cc). Among these genes, the expression of proinflammatory mediators including *IL-12β*, *Hmgb1*, *Cxcl10*, *Ccl2*, and *IL-1β* were downregulated in 3D-MG (Fig. 5Cd).

Sterile inflammation leads to the release of damage-associated molecular patterns (DAMPs), which initiate inflammatory responses through activating DAMP receptors. Among all these DAMP receptors, macrophage-inducible c-type lectin (*Mincle*) is the only one which is reported to be expressed by macrophages and plays a role in the pathogenesis after ischemic stroke [35, 36]. Interestingly, we found a reduction in the expression of *Mincle* in 3D-MG (Fig. 5D). The downregulation of *Mincle* in 3D MSC-infused rats was validated with real-time RT-PCR (Fig. 5E). In order to further validate the effects of 3D MSCs on the expression of *Mincle*, the in vitro model using BV2 microglial cells was used. LPS-activated microglial cells were subjected with the conditioned media of 2D or 3D MSCs and the expression of *Mincle* was determined. Results also showed a significant decrease of *Mincle* expression in 3D MSC-treated microglia (Fig. 5F). These data suggest that Mincle may be involved in 3D MSC-mediated reduced activation of microglia after ischemic injury.

## Discussion

MSCs are proposed as a promising therapy for treatment of ischemic stroke. Numerous preclinical and clinical studies support the non-invasive IV infusion of MSCs in stroke [7–9]. There is a general consensus that the homing and engraftment potential of cultured MSCs is lost after expanding under conventional culture conditions [37]. In this study, we investigated whether 3D culture condition can enhance the homing potential of MSCs. To this end, we transplanted MSCs in MCAO rats and results confirmed enhanced homing of 3D MSCs to the ischemic brain. This may due to the increased expression of chemokine receptor *CXCR4* after 3D culture. *CXCR4* encodes a well-known receptor for chemokine CXCL12, which is mainly produced by damaged neurons after cerebral ischemia [38]. The CXCL12/CXCR4 axis mediates homing of MSCs to the sites of injury [39].

After ischemic stroke, an inflammatory response is stimulated with raised proinflammatory cytokines and reduced anti-inflammatory and trophic factors [40]. The anti-inflammatory properties of MSCs in stroke therapy have been extensively mentioned. MSCs can promote the polarization of macrophages towards an anti-inflammatory phenotype by producing immunosuppressive molecules and metabolites including prostaglandin E2 [41], TSG-6 [42], lactate [43], kynurenic acid [42], and spermidine [44]. MSCs can also prevent the infiltration of monocytes, macrophages, and neutrophils to inflammation sites through the production of TSG-6 [42, 45]. Moreover, MSCs can decrease markers of microglial activation (lower ED1 and Iba1) [21]. However, the underlying molecular mechanisms of MSCs in modulating microglia are not fully elucidated. To this end, we determined the transcriptomic changes of microglia with administration of 2D or 3D MSCs. RNA-seq results showed high similarity of the transcriptome from Sham-MG and 3D-MG, indicating reduced microglial activation by 3D MSCs transplantation. GO biological process analysis implied involvement of downregulated genes in 3D-MG in biological processes including cellular response to LPS, inflammatory responses, immune response, and immune system process. GSEA also demonstrated enrichment of the DEGs in PBS-MG and 2D-MG in immune response. Moreover, the proinflammatory mediators, such as *IL-1β*, *IL-12β*, *Hmgb1*, *Cxcl10,* and *Ccl2* were downregulated in 3D-MG. As for the mechanism of the anti-inflammatory effects of 3D MSCs on microglia, we determined the expression of anti-inflammatory mediators *STC1*, *HGF,* and *TSG-6*. Results showed significant increase of their expression in 3D MSCs. However, whether 3D MSCs exhibited anti-inflammatory effects on microglia through secreting *STC1*, *HGF,* and *TSG-6* need to be further investigated.

**Fig. 6:**
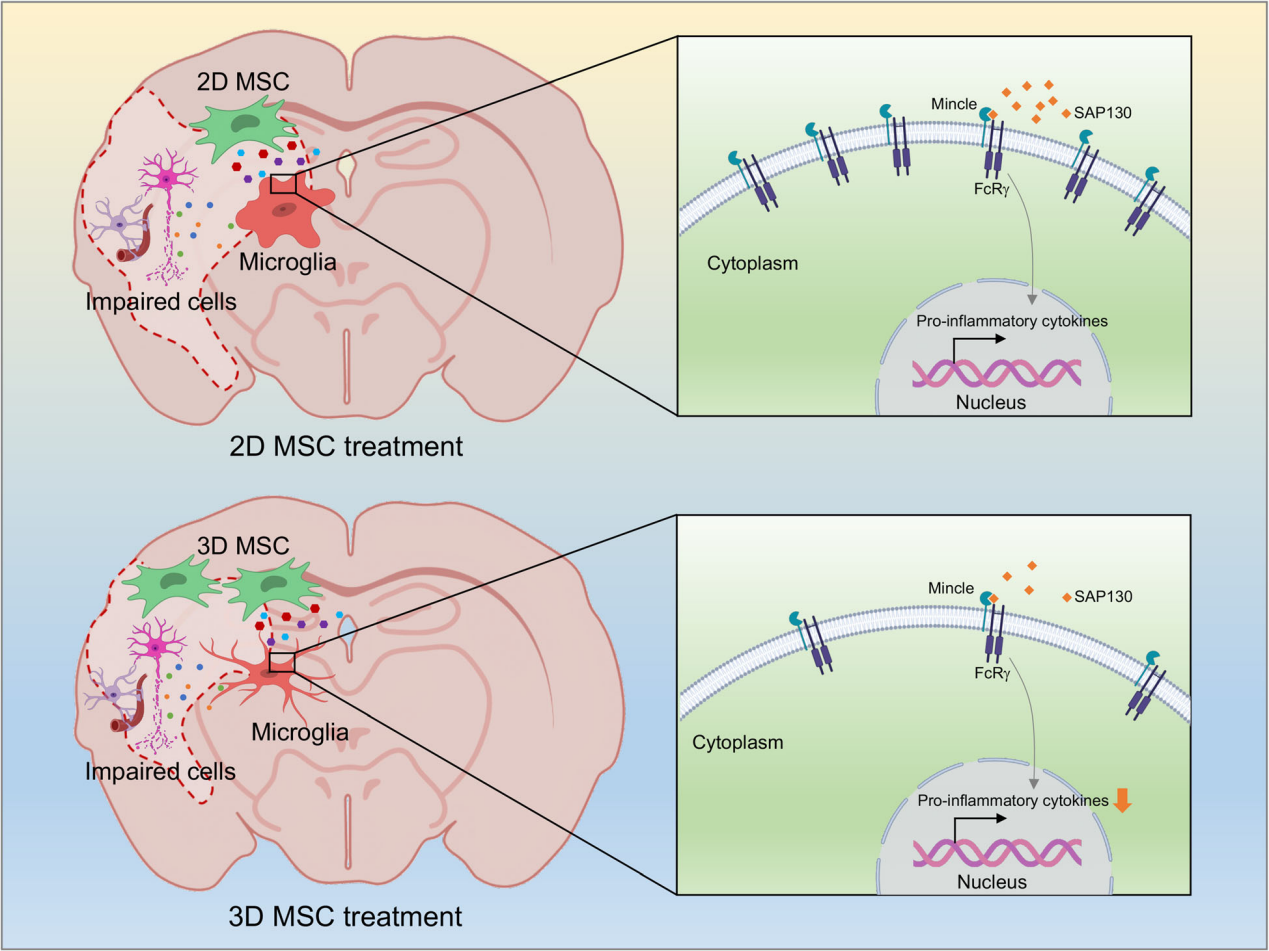
A schematic diagram depicting a potential mechanism of 3D MSC-mediated repair to ischemic brain injury. Cells in the ischemic brain tissue release damage-associated molecular patterns (DAMPs) such as Sin3A associated protein 130 (SAP130), which bind to DAMP receptors such as Mincle on the surface of microglia, mediating increased production of proinflammatory cytokines by the cells. 3D MSCs exhibit enhanced homing and engraftment into the ischemic brain tissue following systemic administration, where they release mediators that decrease the expression of Mincle in microglia, thus reducing their production of proinflammatory cytokines

Sterile inflammation is stimulated by physical or chemical damage which leads to the release of damage-associated molecular patterns (DAMPs). DAMPs initiate an inflammatory response through the activation of pattern recognition receptors, including Toll-like receptors, NOD-like receptors, retinoic acid-inducible gene I-like receptors, C-type lectin receptors, and multiple intracellular DNA sensors [46]. Strikingly, we observed an attenuated expression of a DAMPs receptor, Mincle, in microglia derived from 3D MSCs injected rats. Mincle belongs to C-type lectin receptors and has been implicated in the development of inflammatory diseases. Mincle can induce proinflammatory responses in ischemic stroke model once binding Sin3A associated protein 130 (SAP130), a small ribonucleoprotein released by dying cells [36]. Mincle recognizes β-glucosylceramide, an intracellular metabolite released by damaged cells, and induced the production of proinflammatory cytokines by antigen-presenting cells [47]. The enhanced inflammation caused by the accumulation of β-glucosylceramide can be ameliorated by deletion of *Mincle* [47]. In our in vitro study, we observed a decreased expression of proinflammatory cytokines and Mincle with the treatment of the conditioned media from 3D MSCs. Our results suggest that decreased expression of Mincle in microglia mediated by 3D MSCs may be a cause of reduced inflammation in the injured tissue (Fig. 6).

## Conclusions

In this study, we can conclude that 3D MSCs exhibited enhanced homing ability to the ischemic hemisphere. Moreover, improved therapeutic outcomes were observed in MCAO rats with 3D MSC administration. This may due to enhanced anti-inflammatory properties of 3D MSCs on microglia through reducing the expression of Mincle by microglia. In this sense, 3D MSCs could be exploited as a much safer and efficient alternative for the treatment of immune-mediated disorders.

## Supplementary Information

**Additional file 1: Supplemental Figure 1**. (A) Representative fluorescence-activated cell sorting (FACS) plots showing the population of microglia in brain tissue samples of different treatment groups. Dead cells were excluded with a dead cell marker 7-aminoactinomycin D (7-AAD) and debris by size. (B) The bar graphs depicting the percentage of microglia in all cells. Data are shown as mean ± SEM; n=3 for 2D MSCs group, n=2 for Sham, PBS and 3D MSCs group. **Supplemental Table S1**. Primers for real-time RT-PCR.

## Data Availability

The datasets used and/or analyzed during the current study are available from the corresponding author on reasonable request.

## Abbreviations

3D: Three-dimensional
MSCs: Mesenchymal stem cells
DAMPs: Damage-associated molecular patterns
IV: Intravenous
2D: Two-dimensional
Iba1: Ionized calcium-binding adapter molecule 1
IL-6: Interleukin-6
TSG-6: Tumor necrosis factor-stimulated gene-6
HGF: Hepatocyte growth factor
STC1: Stanniocalcin 1
MCAO: Middle cerebral artery occlusion
mNSS: Modified neurological severity score
DEGs: Differentially expressed genes
NeuN: Neuronal nuclei
GFAP: Glial fibrillary acidic protein
IL-1β: Interleukin-1β
PCA: Principal component analysis
GO: Gene Ontology
LPS: Lipopolysaccharide
GSEA: Gene set enrichment analysis
SAP130: Sin3A associated protein 130
Mincle: Macrophage-inducible c-type lectin
FPKM: Fragments per kilobase of transcript per million mapped reads
