# Supplementary material for: Three-dimensional cultured mesenchymal stem cells enhance repair of ischemic stroke through inhibition of microglia
Source: Stem Cell Res Ther. 2021 Jun 21;12:358. doi: 10.1186/s13287-021-02416-4 (PMC8218508; doi:10.1186/s13287-021-02416-4)
Supplement: Supplementary file 1 — Additional file 1: Supplemental Figure 1. (A) Representative fluorescence-activated cell sorting (FACS) plots showing the population of microglia in brain tissue samples of different treatment groups. Dead cells were excluded with a dead cell marker 7-aminoactinomycin D (7-AAD) and debris by size. (B) The bar graphs depicting the percentage of microglia in all cells. Data are shown as mean ± SEM; n=3 for 2D MSCs group, n=2 for Sham, PBS and 3D MSCs group. Supplemental Table S1. Primers for real-time RT-PCR. [file 13287_2021_2416_MOESM1_ESM.docx]

**SUPPLEMENTARY MATERIAL**

**Supplemental Figure 1.** (A) Representative fluorescence-activated cell sorting (FACS) plots showing the population of microglia in brain tissue samples of different treatment groups. Dead cells were excluded with a dead cell marker 7-aminoactinomycin D (7-AAD) and debris by size. (B) The bar graphs depicting the percentage of microglia in all cells. Data are shown as mean ± SEM; n=3 for 2D MSCs group, n=2 for Sham, PBS and 3D MSCs group.

**Supplemental Table S1. Primers for real-time RT-PCR**

| Gene symbol | Source | Primer sequences |
| --- | --- | --- |
| STC1 | Human | Forward GCAGGAAGAGTGCTACAGCAAG  Reverse CATTCCAGCAGGCTTCGGACAA |
| HGF | Human | Forward GAGAGTTGGGTTCTTACTGCACG  Reverse CTCATCTCCTCTTCCGTGGACA |
| TSG6 | Human | Forward TCACCTACGCAGAAGCTAAGGC  Reverse TCCAACTCTGCCCTTAGCCATC |
| Iba1 | Rat | Forward GCCTCATCGTCATCTCCCCA  Reverse AGGAAGTGCTTGTTGATCCCA |
| CD45 | Rat | Forward CCGTTGTACACCAGAGATGA  Reverse TCCCAAAATCAGTCTGCAC |
| IL-1β | Rat | Forward CACAGCAGCATCTCGACAAGA  Reverse CACGGGCAAGACATAGGTAGCT |
| IL-6 | Rat | Forward GCCCTTCAGGAACAGCTATGA  Reverse TGTCAACAACATCAGTCCCAAGA |
| Mincle | Rat | Forward TGCTACCTTGAAGCATCAGG  Reverse GGTTTTGTGCGAAAAAGGAA |
| Mincle | Mouse | Forward CCAAGTGCTCTCCTGGACGATA  Reverse CTGATGCCTCACTGTAGCAGGA |
